# Time to entry point and distal locking of intramedullary nails: a methodological phantom study comparing biplanar and uniplanar surgical imaging

**DOI:** 10.1186/s12891-022-05130-1

**Published:** 2022-02-24

**Authors:** Peter Ström, Nils P. Hailer, Olof Wolf

**Affiliations:** Department of Surgical Sciences, Orthopaedics, Uppsala University, Uppsala, Sweden

**Keywords:** Biplanar Imaging, Uniplanar Imaging, Intramedullary Nailing, Entry Point, Distal Locking, Radiation

## Abstract

**Background:**

Intramedullary nailing is the method of choice for diaphyseal fractures of the femur and tibia and is also commonly used to treat trochanteric hip fractures. Perioperative imaging is essential for visualising adequate reduction, achieving an optimal entry point (EP) and performing distal locking (DL) of intramedullary nails. This methodological study aims to compare biplanar and uniplanar imaging in some steps of intramedullary nailing.

**Methods:**

We used a biplanar preassembled imaging device (Biplanar™ 600s, Swemac Imaging) and a uniplanar imaging device (Ziehm Solo FD, Ziehm Imaging) to measure procedural and radiation times for antegrade and retrograde femoral and antegrade tibial nailing in fully soft flexible tissue encased legs with radiopaque sawbones (SKU:1515–7-11, Sawbones Europe, Malmö, Sweden). Four orthopaedic surgeons with different levels of experience performed all procedures in all three phantoms with both image techniques in random order, producing in total 12 EPs and nailings with DL with each imaging device. Time to EP, radiation times, time to DL for both devices and the number of swings of the uniplanar device for the two procedures were measured. Comparisons between the biplanar and uniplanar systems with a paired-samples t-test were conducted.

**Results:**

Using the biplanar device, time to optimal EP was shorter for retrograde femoral (26 s (SD15) vs 35 s (SD13), *p* = 0.01) and for antegrade tibial nailing (23 s (SD13) vs 49 s (SD24), *p* = 0.001). No statistically significant differences in time to EP, radiation time or time to DL were found for antegrade femoral nailing. A median of two swings of the uniplanar device was needed to obtain optimal EP for all procedures.

**Conclusions:**

Biplanar imaging slightly but statistically significantly reduced time to EP for retrograde femoral and antegrade tibial nailing in this methodological study comparing biplanar and uniplanar imaging techniques. Biplanar imaging can reduce time and radiation exposure when defining the EP around the knee in intramedullary nailing procedures, but the clinical relevance of these time savings remain to be defined. For antegrade femoral nailing we found no clear benefit with biplanar imaging in the investigated steps of nailing.

## Background

Perioperative imaging is fundamental in orthopaedic trauma surgery, where uniplanar imaging is the method of choice for two-dimensional imaging. When using intramedullary devices for the treatment of femoral and tibial fractures uniplanar fluoroscopy is used to achieve fracture reduction, identify entry points (EP), and insert locking screws. Adequate identification of EPs is crucial to attain correct implant positioning and, as regards retrograde femoral and antegrade tibial nailing, to avoid intraarticular chondral defects [1, 2].

Although widely adopted, uniplanar fluoroscopy can be perceived as inefficient because of the need to rotate the C-arm multiple times to obtain orthogonal images for all intervention steps. Simultaneous use of two C-arms to achieve good anteroposterior (AP) and lateral imaging without repositioning the fluoroscopy unit has been described [3–5]. This approach potentially reduces the need for swinging the C-arm and may reduce the risk of surgical site contamination [4]. In Scandinavian countries, prefabricated biplanar imaging machines were introduced in the 1970s and are now widely used in orthopaedic surgery. Some other countries using biplanar imaging are Poland, China, Taiwan and Japan. A preassembled biplanar device avoids the bulky establishment of two separate C-arms to achieve simultaneous biplanar imaging, with independent foot pedals for the respective images. Moreover, biplanar devices offer functionality and facilitate EP determination and distal locking (DL) [6].

However, it is unclear whether using a biplanar device reduces operating and radiation times compared to a conventional uniplanar device. Therefore, this methodological phantom study aims to compare time to EP and DL during ante- and retrograde intramedullary nailing of the femur and antegrade nailing of the tibia, using either preassembled biplanar imaging (Biplanar™ 600s, Swemac Imaging, Swemac Medical Imaging Devices, Täby, Sweden) or a uniplanar C-arm (Ziehm Solo FD, Ziehm Imaging, Nuremberg, Germany) with repositioning.

## Methods

**Fig. 1:**
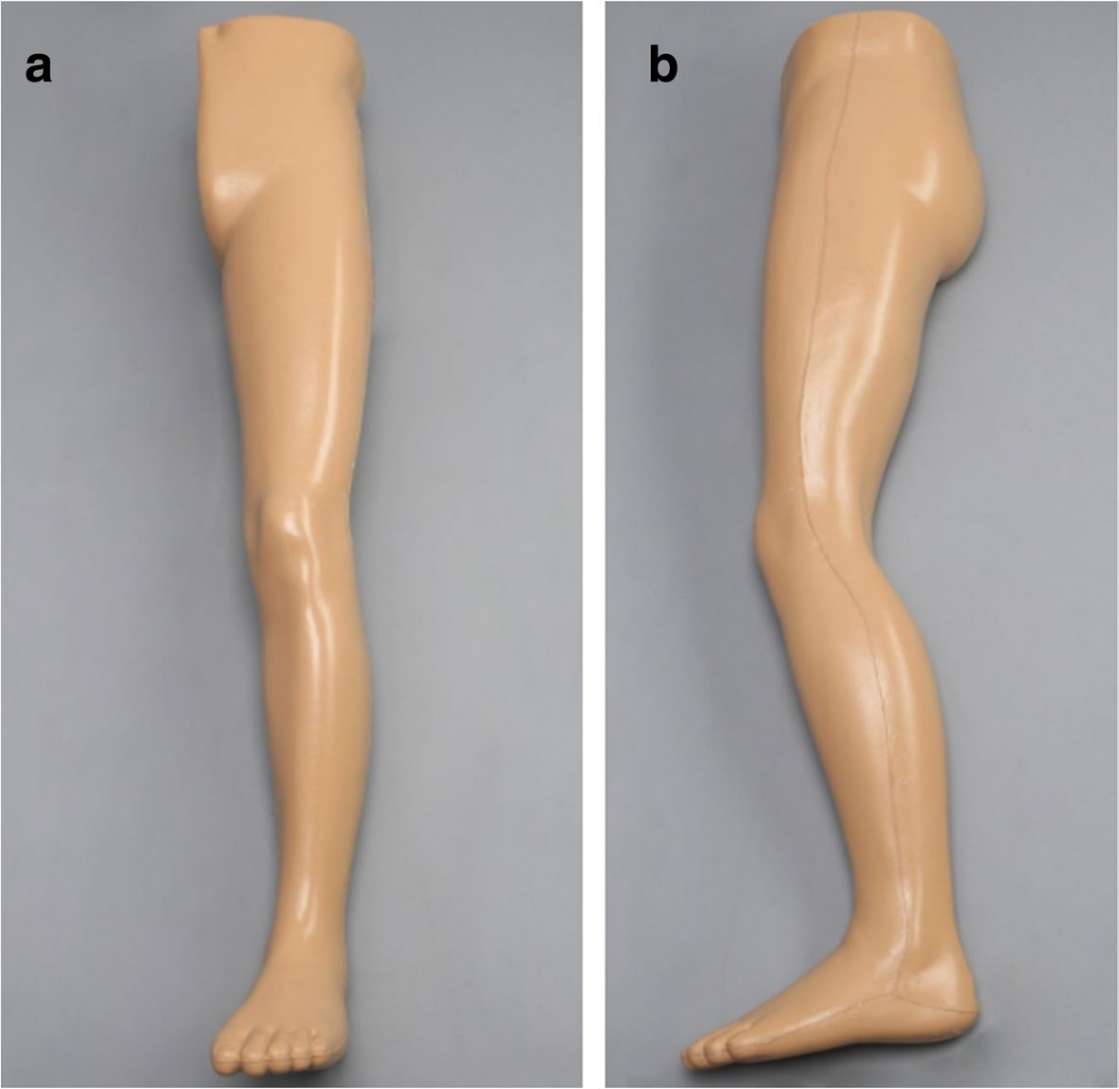
Fully soft tissue encased sawbones

**Fig. 2:**
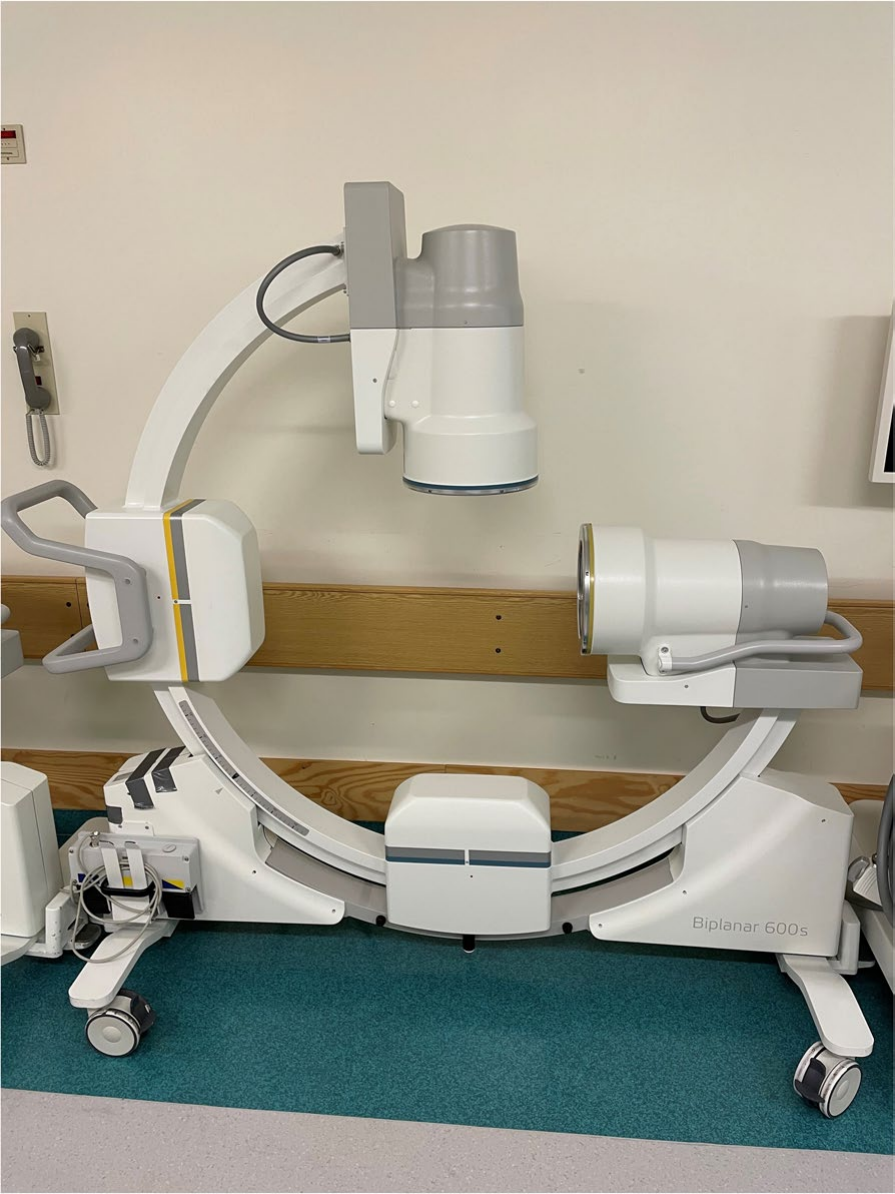
Biplanar imaging (Biplanar™ 600 s, Swemac Imaging) used in the study

**Fig. 3:**
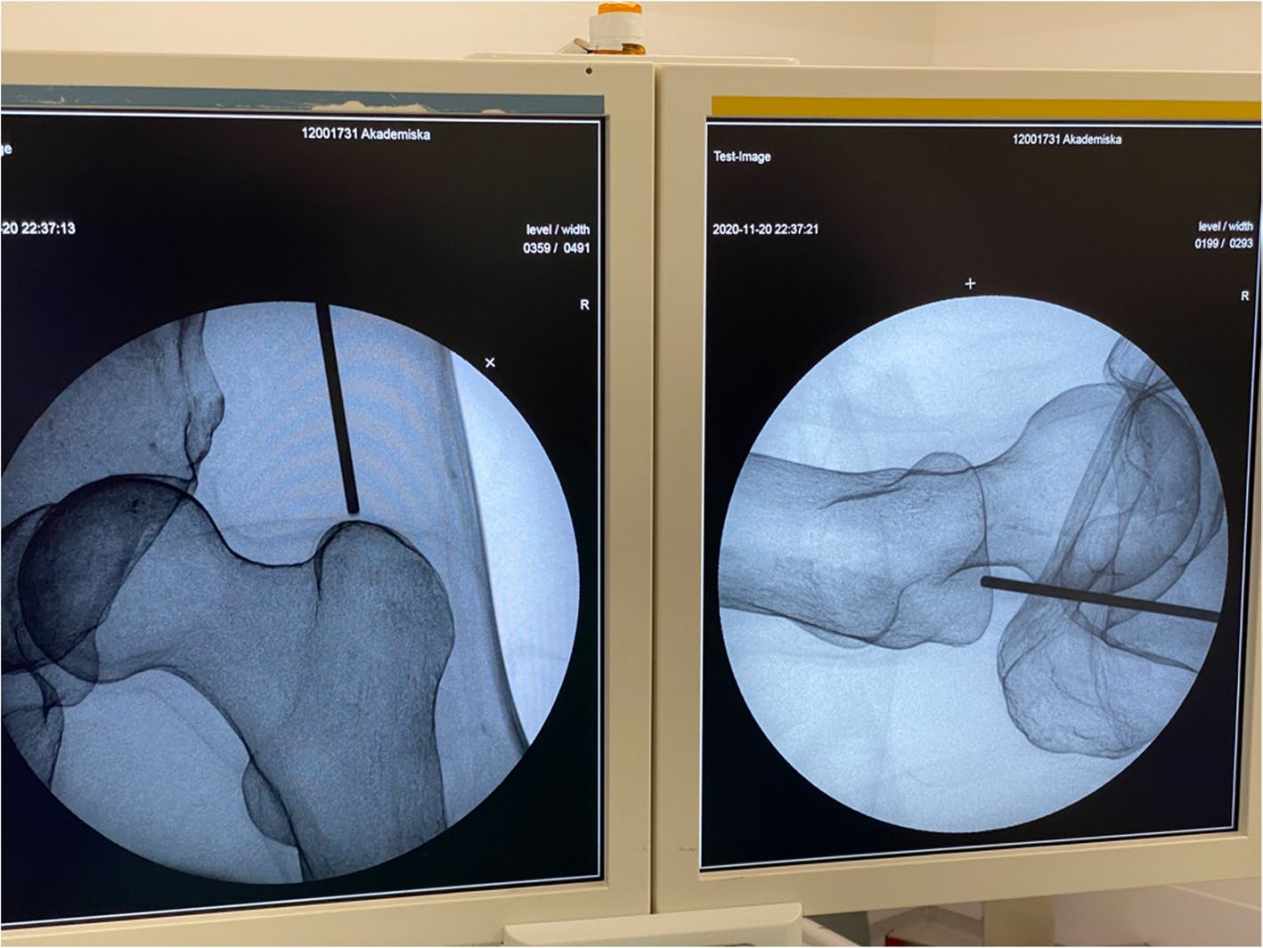
Hip entry point under biplanar imaging

**Fig. 4:**
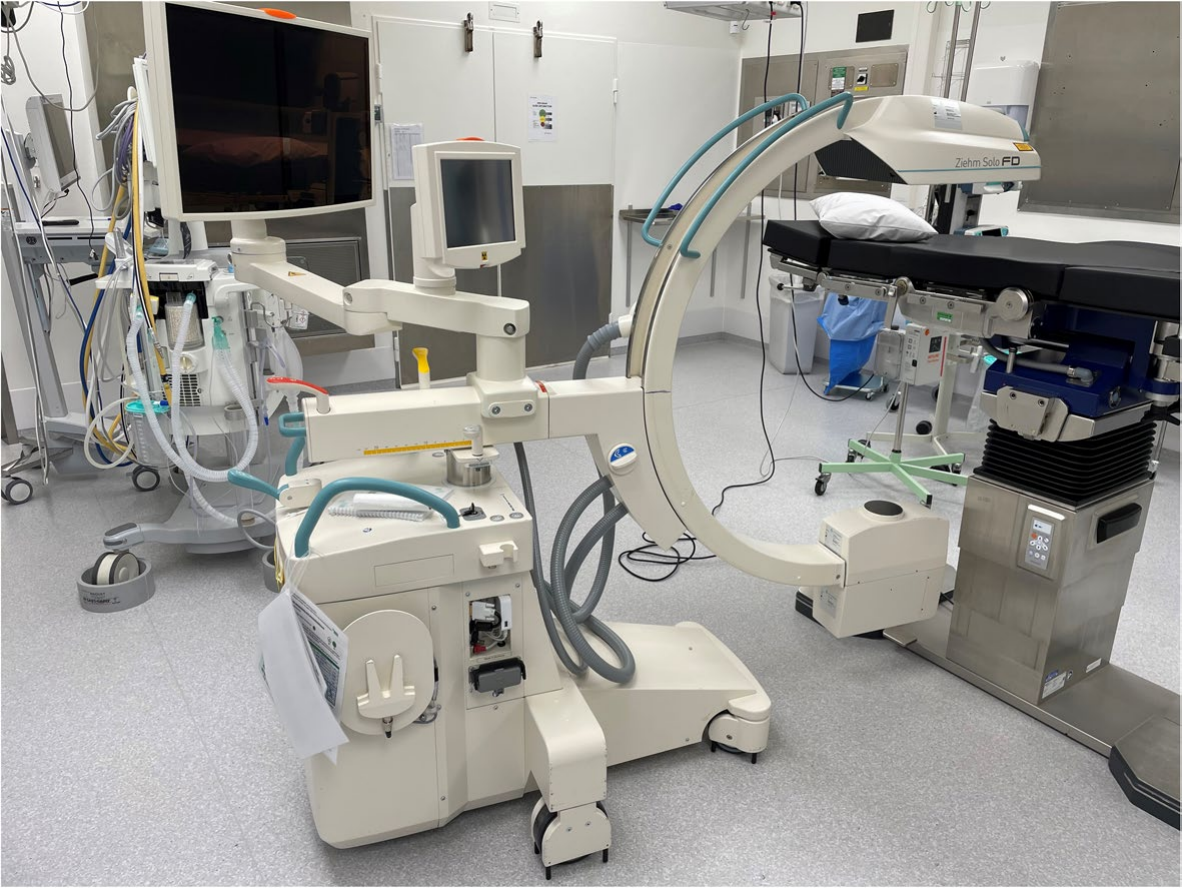
Uniplanar imaging (Ziehm Solo FD, Ziehm Imaging) used in the study

This study applies a methodological framework to compare biplanar and uniplanar imaging for the times needed to conduct key steps while performing intramedullary nailing. We used intramedullary nailing of the femur (Proximal Femoral Nail Antirotation, PFNA, DePuy Synthes, Oberdorf, Switzerland) and tibia (Expert Tibial Nail, DePuy Synthes, Oberdorf, Switzerland) on fully soft tissue encased legs with radiopaque sawbones (SKU:1515–7-11, Sawbones Europe, Malmö, Sweden) (Fig. 1). Biplanar imaging was done with a preassembled device (Biplanar™ 600 s, Swemac Imaging, Swemac Medical Imaging Devices, Täby, Sweden) containing two perpendicular independent image sources mounted in a G-stand (Fig. 2). The device allows for simultaneous AP and lateral projections displayed on separate screens (Fig. 3). For uniplanar imaging, a C-arm (Ziehm Solo FD, Ziehm Imaging, Nuremberg, Germany) was used (Fig. 4). This device is slightly smaller but requires repositioning or swinging the C-arm to obtain AP and lateral views.

**Fig. 5:**
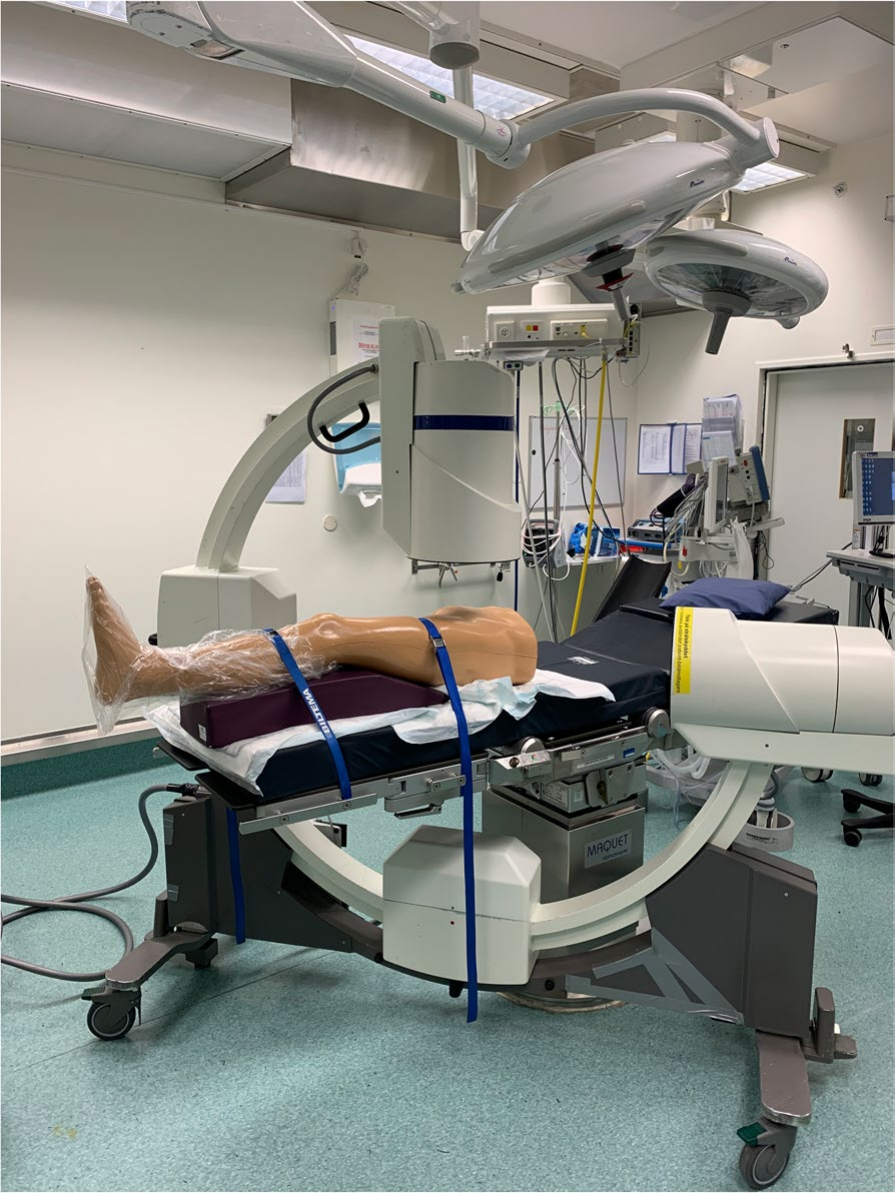
Securely positioned sawbone with biplanar imaging device

The encased legs were securely fastened and positioned on a radiolucent fracture table (Fig. 5). Three orthopaedic trauma surgeons with varying experience (1, 10 and > 15 years post-training) and one orthopaedic trainee performed the procedures. Each surgeon executed each of three procedures: Antegrade insertion of the intramedullary femoral nail including DL, identification of EP for retrograde femoral nail and antegrade insertion of the tibial nail including DL. Each of the four surgeons performed each procedure three times on different sawbones with uniplanar imaging and three times with biplanar imaging, producing 12 sets of procedures. The four surgeons conducted the procedures in random order and times (in seconds) for procedures and radiation time were recorded. The fully soft tissue encased leg with radiopaque sawbones was dressed in surgical drapes. We measured time from skin incision to positioning of a guidewire at the tip of the greater trochanter as the optimal EP (aligned with the intramedullary femoral canal) for the PFNA, time elapsed from skin incision to bicortical drilling through the nail in preparation for one DL screw (static) through a long PFNA femoral nail, time from skin incision to guidewire positioning at the optimal EP (aligned with the intramedullary femoral canal and in extension with Blumensaat’s line) for the retrograde femoral nail, time from skin incision to guidewire positioning at the optimal EP (in the middle of the intercondylar notch, aligned with the axis of the intramedullary tibial canal and on the anterior edge of the proximal tibia) for the Expert Tibial Nail using a suprapatellar approach and time elapsed from skin incision to bicortical drilling through the nail in preparation for one DL screw (static). We used three sawbones and performed the distal drilling at different levels by adjusting the nail position to achieve an untouched area. DL was performed with the uniplanar imaging in the lateral position and time measurement was initiated once an optimal image had been obtained. The image intensifiers were controlled by foot control by the orthopaedic surgeon. Repositioning of uniplanar imaging was done by an assistant theatre nurse. The number of performed swings of the uniplanar imaging and the number of missed drillings (anterior or posterior to nail) for DL were noted. Accurate positioning was determined by fellow orthopaedic surgeons.

### Statistical analysis

Calculation times to identify EP, bicortical drilling for the DL screw and radiation times are reported in seconds using means and standard deviations (SD) and the number of swings and missed drillings as medians and range. Differences between biplanar and uniplanar imaging were compared using the paired-samples t-test for each. Individual values for time to EP and time to DL were plotted in grouped scatter plots. All statistical calculations were made with SPSS version 27 and *P* < 0.05 was considered statistically significant.

## Results

**Fig. 6:**
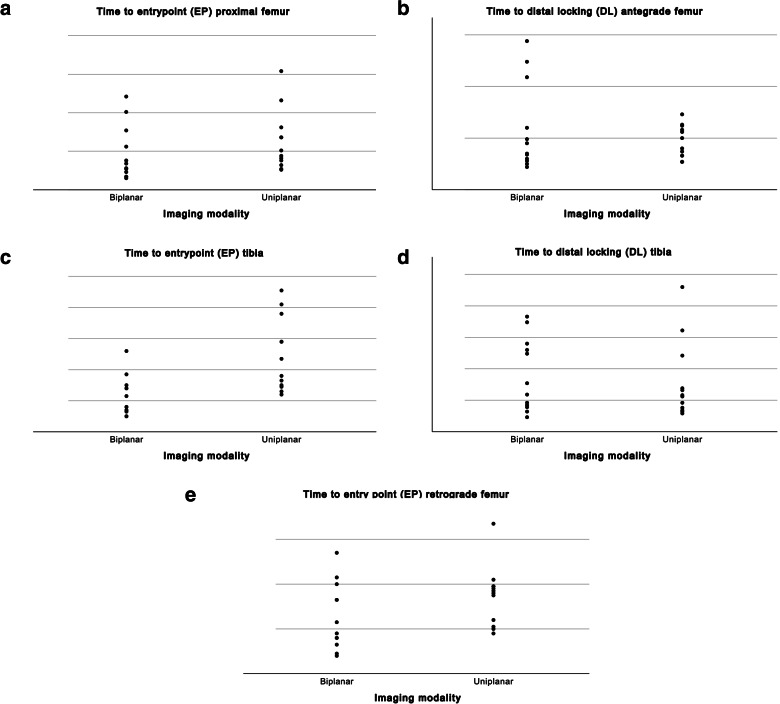
Individual values for 12 attempts of time (seconds) to entrypoint and distal locking in antegrade and retrograde femoral nailing and antegrade tibial nailing grouped by biplanar and uniplanar imaging device

The comparison for all procedures is presented in Table 1. All 12 individual times to EP and DL for each procedure are visualised in Fig. 6.

**Table 1:** Mean time to entrypoint (EP) in seconds (SD; range), mean radiation time in seconds (SD; range), median number of swings of C-arm (range), and median number of missing attempts for distal locking (DL) (range). *P* value < 0.05 considered significant

	Biplanar	C-arm	*P* value
Antegrade Femur			
Time EP	46 (35; 15–121)	62 (39; 26–154)	0.16
Radiation EP	11 (6; 7–23)	14 (7; 4–25)	0.34
Swings EP	na	2 (1–6)	
Time DL	59 (42; 22–144)	47 (16; 27–73)	0.35
Radiation DL	18 (12; 7–44)	15 (5; 8–23)	0.49
Swings DL	na	lateral position	
Missing hole DL	0 (0–2)	0 (0–1)	
Retrograde Femur			
Time EP	26(15; 8–54)	35 (13; 18–67)	0.01
Radiation EP	8 (5; 2–16)	10 (5; 5–23)	0.30
Swings EP	na	2 (2–3)	
Antegrade Tibia			
Time EP	23 (13; 10–52)	49 (24; 24–91)	0.001
Radiation EP	8 (3; 4–13)	14 (6; 7–29)	0.007
Swings EP	na	2 (2–6)	
Time DL	89 (58; 23–183)	78 (62; 29–230)	0.61
Radiation DL	26 (15; 7–55)	24 (16; 9–56)	0.76
Swings DL	na	lateral position	
Missing hole DL	1 (0–2)	0 (0–2)

### Antegrade nailing of the femur

No statistically significant difference was found in time to EP between biplanar and uniplanar imaging. Mean time to EP was 46 s (SD35) for biplanar and 62 s (SD39) for the uniplanar device. There was also no statistically significant difference in radiation times. A median of two swings of the C-arm was required to achieve an optimal EP into the proximal femur. Time to DL did not differ between bi- and uniplanar imaging, 59 s (SD42) versus 47 s (SD16). Missing the DL hole was rare for both devices.

### Retrograde nailing of the femur

A statistically significantly shorter time to EP was observed with biplanar imaging (26 s (SD15)) compared to uniplanar imaging (35 s (SD13)). Radiation times did not differ between the two imaging modes. A median of two swings was used to achieve optimal EP for retrograde femoral nailing.

### Antegrade nailing of the tibia

Time to EP was statistically significantly shorter for biplanar imaging, 23 s (SD13) vs 49 s (SD24). Also, radiation time for finding the optimal EP was statistically significantly shorter with biplanar imaging, 8 s (SD3) vs 14 s (SD6). A median of two swings of the uniplanar device was required to achieve an optimal EP into the proximal tibia. Time to DL for the distal tibia and radiation time for DL did not differ significantly between bi- and uniplanar imaging. No relevant difference between imaging modes in radiation times was detected. A median of one missed DL drilling was observed for biplanar imaging compared to zero missed for uniplanar imaging.

### Subsequent procedures and surgeon experience

In this methodological setting times were generally fast for all 4 surgeons. We did not experience a clear trend towards faster times regarding subsequent attempts for any of the surgeons. Each surgeon had some trouble at some of the procedures, so times went in both directions without any clear trend. More missed DL screws were noted in later attempts as surgeons tried to do it faster. The two more experienced surgeons were generally faster than the more inexperienced for all procedures (see individual values, Fig. 6).

## Discussion

### Principal findings

This study reports the timing of different steps of an intramedullary nailing procedure of the femur or tibia. The main finding of this study is that the use of a biplanar device slightly reduced time to identify EP for tibial nailing and retrograde femoral nailing. A reduction in radiation time was found only in time to EP for the tibia but not for the femur. There was a tendency towards shorter times for DL for both antegrade femur and tibia with uniplanar imaging, although not statistically significantly so, when compared to biplanar imaging.

### Agreement and disagreement with the literature

The choice of correct EPs is critical in the stabilisation of femoral or tibial fractures with intramedullary nails. Indeed, failure to identify the right EP can cause fracture malalignment and iatrogenic fracture, such as has been described for lateral femoral nails [1]. Not all EPs are equal, as was shown when comparing fluoroscopy times required for the medial posterior and the lateral anterior trochanteric EP when using the PFNA device [7]. In antegrade femoral nailing trochanteric overhang can obstruct the approach to the piriformis fossa for nail insertion in about 25% of the patients [8]. Additionally, chondral defects in the knee that may be more frequent when using suprapatellar than parapatellar approaches can be avoided using the correct anatomical location of EPs [2].

Although using a uniplanar device, commonly called a C-arm, and manual nail insertion and reaming during intramedullary nailing are clinically the most common method, alternative techniques (e.g., biplanar and robotic devices) have been explored [4, 9]. A combination of fluoroscopy and navigation has been described, but nonorthogonal imaging reduced the accuracy of EP identification, even in the presence of navigation [10].

### Strengths and weaknesses in relation to other studies

We sought to investigate whether a biplanar device can reduce the time required to identify adequate EPs and the time for DL during intramedullary nailing of the femur or tibia.

We have not taken preoperative positioning and draping of real patients into consideration in this methodological study. In closed reduction in the fracture table it often takes several swings of the C-arm to confirm reduction. When draping the C-arm, it must swing freely so as not to contaminate the operating field or get stuck and frustrate the surgeon. Biplanar imaging is preassembled and both the reduction manoeuvre and the operating procedure can be performed without repositioning. For DL, the image sources are advanced distally and a fast and reliable technique for DL using biplanar imaging has been widely adopted [6]. Both AP and lateral image sources are draped before the procedure. There is even room for the surgeon to introduce long femoral nails between the patient and the lateral image source.

Our clinical experience is that nail procedures are easier and faster with biplanar imaging, although no objective data supports this, and, to our knowledge, there are no comparative studies to confirm this view. The simultaneous use of two C-arms reduced the number of attempts to achieve optimal pin position in percutaneous pinning of proximal humerus fractures while reducing excess exposures and potential surgical field contamination [11]. In the repair of supracondylar humerus fractures there was no difference in fluoroscopy time or radiation dose for biplanar or uniplanar imaging using one or two C-arms [12]. There was no significant difference in surgery times for slipped capital femoral epiphysis, but the screw positioning was better in the biplanar modus using two C-arms [3]. Both these studies are clinical, comparing surgery times and radiation doses for full procedures. Our methodological study using soft-tissue ensheathed saw bone phantoms examined steps of a full procedure using a preassembled biplanar imaging device compared to a C-arm. When using biplanar imaging, surgeons with different levels of expertise were slightly quicker in locating the EP in the tibia and for retrograde femoral nailing, but differences were small. We found no difference in DL time; however, the time began with the uniplanar device arranged in a perfect lateral position. Such a configuration will introduce swinging of the uniplanar device with sterile drapes that can hinder free movement in the clinical setting. It is also time-consuming to find the correct true lateral projection in order to safely perform DL. Unfortunately, we did not time this set up which would have resulted in longer times for DL and thus be advantageous for the biplanar device in a direct comparison. The use of biplanar imaging for intramedullary nailing of tibia and femur has been recommended to overcome the drawbacks of uniplanar imaging, i.e. time consumption and risks of contamination [4].

The inevitable swinging of the uniplanar C-arm confers an increased risk of surgical field contamination [4, 11]. Contamination of the surgical field was linearly increased with increased cycles/swings of the C-arm with all areas contaminated after 15 cycles in a methodological study using fluorescent powder contaminant [13]. The area closest to the C-arm had the most pronounced contamination. Contamination of C-arm drapes are common, they are correlated to swinging of the C-arm and occur early during procedures [14]. The surgeon-operated mini C-arm showed contamination of the drapes in 70% of the cases [15]. Newer draping techniques of the C-arm compared to older seem to decrease contamination [16, 17]. A future comparative study between uniplanar and biplanar imaging regarding surgical field contamination might be warranted.

Our study's methodological arrangement does not consider the full clinical reality of bleeding and drapes that move while repositioning, becoming immovable when trying to swing back. On the other hand, we found that both techniques work equally reliable in the hands of experienced as well as inexperienced orthopaedic surgeons. DL with biplanar imaging, using the technique described by Granhed in 1998 [6], is fast but requires some practising before the technique is fully understood and works in the surgeon's hands. The wide range of procedural times for DL in the distal femur nicely illustrates this fact.

The total time for the procedure is not fully accounted for by our methodological study measuring procedural steps, given that DL was measured after the C-arm was tilted to the desired lateral position for both the distal femur and the distal tibia. With the biplanar device, one slides it down to the right location and then both AP and lateral images are displayed without much further adjustment. Based on our clinical experience with biplanar imaging, performing two or three DL screws with drilling and inserting the screws is much faster. In this methodological setting we measured the time for only one DL screw for each position.

Biplanar imaging is sometimes criticised for obstructing the surgeon’s range of action in accessing the operating field. The clinical experience is that when nailing the femur and tibia, it is rarely necessary to move the biplanar image sources other than along the extremity. One can preoperatively widen the distance of the lateral image sources to access the proximal femur allowing the reamer and even a long nail to be introduced.

The antegrade femoral nailing in our study was conducted by inserting the PFNA via the tip of the greater trochanter, an EP that is suggested to be advantageous (in terms of fluoroscopy times) over entering via the piriformis fossa [18]. Our choice of a suprapatellar EP for antegrade tibial nailing is consistent with current clinical practice, although this approach can cause cartilage and anterior cruciate ligament defects despite good fluoroscopic control [2]. The suprapatellar approach also results in greater EP accuracy compared to the parapatellar approach [19].

### Strengths and weaknesses

One strength of this study is that we used the same sawbones with the same soft tissue envelope to compare times for different steps of the intramedullary nailing procedure. Four surgeons with varying levels of experience performed the procedures in random order and repeated all procedures three times for both image sources. These steps, however, are somewhat synthetic compared to the clinical setting. Furthermore, the need for imaging certain steps is potentially reduced in the not-so-obese sawbones or when there is no bleeding blurring the surgeon’s view. We did not measure a full intramedullary nailing procedure; nor did we take the repositioning into account for total procedural time. The set up for a true lateral projection with the uniplanar device was not timed, which is a drawback for the comparison of times to DL. The number of swings was measured but only for the uniplanar C-arm.

## Conclusion

The use of biplanar imaging slightly reduces the time to identify the proper EPs around the knee for retrograde femoral and antegrade tibial intramedullary nailing, although this time saving may not be clinically relevant. We also noted a concomitant reduction in radiation time to identify EPs in the tibia, but absolute differences in time were, again, small. We found no statistically significant or clinically relevant reduction in the time to identify the optimal EP in the greater trochanter or the time spent for DL during antegrade intramedullary nailing of the femur or tibia. Taken together, our findings suggest that the use of biplanar imaging may slightly reduce operating time and radiation exposure during defined steps of retrograde femoral and antegrade tibial intramedullary nailing, but that clinical benefits remain to be determined.

## Data Availability

The datasets generated and/or analysed during the current study are available from the corresponding author on reasonable request.

## Abbreviations

AP: Anteroposterior
DL: Distal locking
EP: Entry point
PFNA: Proximal Femoral Nail Antirotation
SD: Standard deviations
